# Identifying Balanced Chromosomal Translocations in Human Embryos by Oxford Nanopore Sequencing and Breakpoints Region Analysis

**DOI:** 10.3389/fgene.2021.810900

**Published:** 2022-01-18

**Authors:** Zhenle Pei, Ke Deng, Caixai Lei, Danfeng Du, Guoliang Yu, Xiaoxi Sun, Congjian Xu, Shuo Zhang

**Affiliations:** ^1^ Shanghai Ji Ai Genetics and IVF Institute, Shanghai Key Laboratory of Female Reproductive Endocrine Related Diseases, Obstetrics and Gynecology Hospital of Fudan University, Shanghai, China; ^2^ Chigene (Beijing) Translational Medical Research Center Co. Ltd., Beijing, China

**Keywords:** balanced translocation, preimplantation genetic testing, long-read sequencing, breakpoint PCR, haplotype linkage analysis

## Abstract

**Background:** Balanced chromosomal aberrations, especially balanced translocations, can cause infertility, recurrent miscarriage or having chromosomally defective offspring. Preimplantation genetic testing for structural rearrangement (PGT-SR) has been widely implemented to improve the clinical outcomes by selecting euploid embryos for transfer, whereas embryos with balanced translocation karyotype were difficult to be distinguished by routine genetic techniques from those with a normal karyotype.

**Method:** In this present study, we developed a clinically applicable method for reciprocal translocation carriers to reduce the risk of pregnancy loss. In the preclinical phase, we identified reciprocal translocation breakpoints in blood of translocation carriers by long-read Oxford Nanopore sequencing, followed by junction-spanning polymerase chain reaction (PCR) and Sanger sequencing. In the clinical phase of embryo diagnosis, aneuploidies and unbalanced translocations were screened by comprehensive chromosomal screening (CCS) with single nucleotide polymorphism (SNP) microarray, carrier embryos were diagnosed by junction-spanning PCR and family haplotype linkage analysis of the breakpoints region. Amniocentesis and cytogenetic analysis of fetuses in the second trimester were performed after embryo transfer to conform the results diagnosed by the presented method.

**Results:** All the accurate reciprocal translocation breakpoints were effectively identified by Nanopore sequencing and confirmed by Sanger sequencing. Twelve embryos were biopsied and detected, the results of junction-spanning PCR and haplotype linkage analysis were consistent. In total, 12 biopsied blastocysts diagnosed to be euploid, in which 6 were aneuploid or unbalanced, three blastocysts were identified to be balanced translocation carriers and three to be normal karyotypes. Two euploid embryos were subsequently transferred back to patients and late prenatal karyotype analysis of amniotic fluid cells was performed. The outcomes diagnosed by the current approach were totally consistent with the fetal karyotypes.

**Conclusions:** In summary, these investigations in our study illustrated that chromosomal reciprocal translocations in embryos can be accurately diagnosed. Long-read Nanopore sequencing and breakpoint analysis contributes to precisely evaluate the genetic risk of disrupted genes, and provides a way of selecting embryos with normal karyotype, especially for couples those without a reference.

## Introduction

As one of the most prevalent genomic structural rearrangements, balanced translocations occur during the process of genetic material between two separate chromosomes interchanging. Regardless of parental age, balanced translocations are frequently linked to infertility. The overall prevalence of balanced translocations is 0.25% in the general population (Jacobs et al., 1992), 1.1% in patients with infertility (Clementini et al., 2005), 4.5% in patients with a history of recurrent abortion (Sugiura-Ogasawara et al., 2004), and 9.2% in couples with more than three first-trimester abortions (Sampson et al., 2004). Carriers with balanced translocations are usually phenotypically healthy, but they suffer from a high rate of imbalanced gametes due to aberrant meiotic segregation (Ford and Clegg 1969; Brandriff et al., 1986). Additionally, balanced chromosomal translocations are capable of inducing duplications, microdeletions and disruption of genes related to infertility (Donker et al., 2017; Schilit et al., 2020). These carriers may sustain higher probability of infertility, repeated spontaneous miscarriages, and chromosomally defective offspring (Campbell et al., 1995).

Preimplantation genetic testing (PGT) offers an effective way for reciprocal translocation carriers to reduce the risk of pregnancy loss due to abnormal chromosomal segregation. PGT can be divided into three categories, namely PGT for aneuploidy (PGT-A), PGT for monogenic (PGT-M), and PGT for structural rearrangement (PGT-SR). Breakpoint sequences cannot be recovered by traditional analytical techniques, such as fluorescence *in situ* hybridization (FISH) (Schluth-Bolard et al., 2013). There is an urgent demand for innovative techniques to precisely distinguish breakpoints or linkage polymorphism markers for embryo diagnosis and genetic risk assessment of carriers. Multiple techniques have been developed to identify copy numbers variants (CNVs) of chromosome fragments, e.g. next-generation sequencing (NGS) (Chow et al., 2018), quantitative polymerase chain reaction (qPCR) (Cimadomo et al., 2018a), single nucleotide polymorphism microarray (SNP array) (Treff et al., 2011a), and array comparative genomic hybridization (aCGH) (Fragouli et al., 2011). However, none of these genetic methods can be employed to discriminate between the carrier and translocation-free embryos. In recent years, new PGT approaches have achieved significant advancement. Owing to preimplantation genetic haplotyping (PGH), translocation carriers can be accurately distinguished from noncarriers with genome-wide haplotype linkage analysis (Zhang et al., 2017; Zhang et al., 2021). Mapping Allele with Resolved Carrier Status (MaReCs) was successfully performed on 96 human embryos from 13 reciprocal translocation carriers and 12 Robertsonian translocation carriers (Xu et al., 2017). Hu, L *et al.* diagnosed 13 carrier blastocysts from 15 balanced blastocysts with the aid of NGS following micro-dissecting junction region preimplantation genetic diagnosis (MicroSeq-PGD). Moreover, they confirmed that this technique could be employed to discriminate reciprocal translocation carriers (Hu et al., 2016).

As a high-throughput sequencing or massively parallel/deep sequencing technology, NGS has been proven to be revolutionary in the genomic age. SNPs and indels are frequently observed with short-read sequencing (SRS), in which reads with the typical length of 100–200 bp proves to be adequate (Eggertsson et al., 2017). However, SRS complicates the genotyping and characterization of structural rearrangements, and the number of chromosomal rearrangements discovered per person in large-scale SRS studies has been restricted to 2,000–11,000 (Sudmant et al., 2015; Abel et al., 2020). Moreover, NGS and array-based methods also fail to identity noncarrier from euploid carrier embryos in PGT-SR cycles, or to find the position of breakpoint junctions (Tsiatis et al., 2010). By contrast, long-read approaches could provide the read length that is well-qualified for breakpoint identification (Goodwin et al., 2016). Long-read sequencing (LRS) provided by Oxford Nanopore Technologies (ONT) and PacBio single molecular real-time (SMRT) sequencing have ushered in a broad prospect of chromosomal aberration genotyping and phasing in recent years (Chaisson et al., 2015; Deamer et al., 2016; Jain et al., 2018). Those readings that are 10 kb or longer on average may be determined by LRS, which conduces to improving the detection of structural variations (SVs) (Lu et al., 2016). As a result, long reads allow for the traversing of complicated or repetitive sections with a single continuous read, thus removing the ambiguity regarding the position or size of genomic components. Notably, LRS is more sensitive and accurate than SRS in mapping SVs across the genome. In a study of LRS at the population scale, more than 22,636 SVs were identified per individual, and this size was three to five times more than that observed in SRS data (Beyter et al., 2021). This benefit is especially noticeable in repeat regions, such as tandem-repeat (TR) regions (Collins et al., 2020). Nanopore sequencing, originally proposed in 2012, is a single-molecule LRS technique capable of directly mapping the DNA structure of a native single-stranded DNA (ssDNA) molecule (Pennisi 2012). When whole-exome sequencing fails to differentiate the breakpoint area in Alu elements, Nanopore sequencing discovers a 7 kb segment deletion in *G6PC* gene, which is related to glycogen storage disorder type Ia (Miao et al., 2018). Furthermore, another widespread long-read technology, PacBio SMRT, can be employed to identify integrated nucleotides based on the fluorescence of the nucleotide released after phosphate chain cleavage (Rhoads and Au 2015). The SMRT sequencing has been successfully employed to screen out a 2184 bp chromosomal deletion in *PRKAR1A* gene, which is one of its first clinical applications in detecting *de novo* structural aberrations in patients *via* LRS (Merker et al., 2018). With the advancement of bioinformatics tools, single-molecule LRS has established itself as a cutting-edge method for PGT-SR cycles of balanced aberration carriers with no need for aneuploid embryos or samples from immediate family as references, which bridges gaps between existing reference assemblies (Hu et al., 2019).

In this study, whole-genome sequencing was implemented in two translocation carriers with LRS provided by the ONT. Besides, PCR and Sanger sequencing were adopted to confirm the discovery of reciprocal translocation breakpoints. In addition, the junction-spanning PCR and haplotype linkage analysis combined with comprehensive chromosomal screening (CCS) were applied to detect biopsied cells of blastocysts. This genetic method, based on Nanopore sequencing data from two carriers, established the groundwork for the larger-scale application of PGT-SR, which promotes the investigation of the SNP allele frequency spectrum and even contributes to the exploration in genomic areas that SRS technologies have not reached.

## Materials and Methods

### Study Design

**FIGURE 1:**
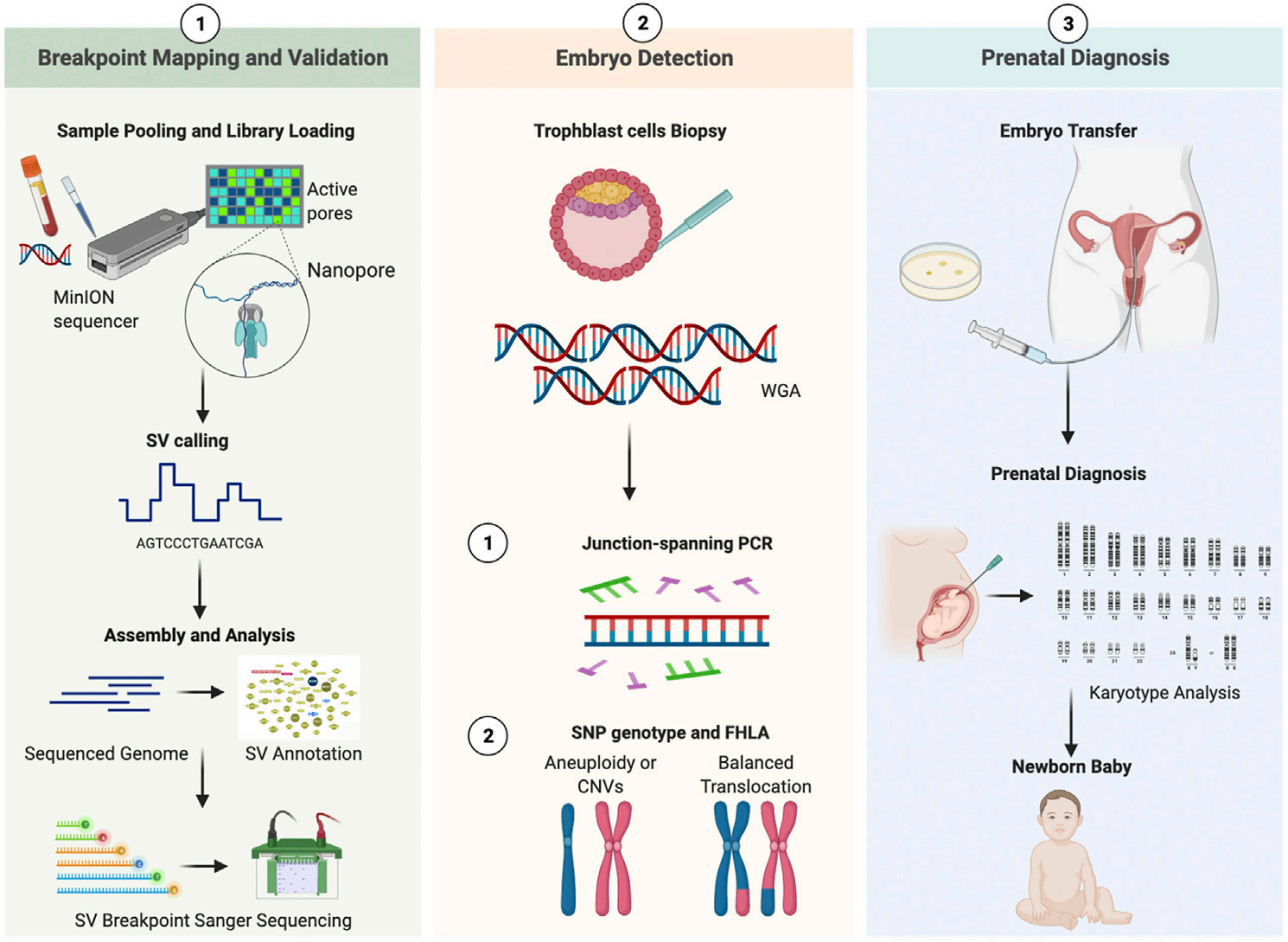
Working pipeline of the study. Legend: The workflow contains three phases: breakpoint mapping and validation, embryo detection, prenatal diagnosis. The procedure is universal for both patients. SV, structural variation; WGA, whole genomic amplification; CNV, copy Number Variation; SNP, single nucleotide polymorphism; FHLA, family haplotype linkage analysis.

This study consisted of three sections (Figure 1). In the preclinical section 1, the parental blood sample was analyzed in an attempt to accurately decipher translocation breakpoints based on long-read, Nanopore sequencing data, and the results were validated by PCR and Sanger sequencing. The outline was generated with BioRender. In the clinical section 2, the study of whole genome amplified (WGA) DNA of embryos was performed, followed by breakpoint PCR and haplotype analysis. In the clinical section 3, the above-mentioned results were compared with those from prenatal diagnosis.

### Patients Recruited

Two patients with cytogenetically confirmed chromosomal translocation who underwent assisted reproductive technologies (ART) were enrolled. Both patients experienced repeated miscarriages. The informed consent forms of both patients were obtained. The karyotype of patient 1 was 46, XX, t(1,2)(1q44; 2q31). While that of patient 2 was 46, XY, t(12; 14)(p13; q24). The karyotype analysis of these two carriers’ parents was also performed. 10 ml of peripheral blood sample was collected from both couples and their family members during recruitment.

### High-Resolution Breakpoint Mapping by Long-Read Nanopore Sequencing

According to the protocols of Oxford Nanopore, UK, after genomic DNA (5 μg) of samples passed the quality inspection, the BluePippin (Sage Science, MA, United States) automatic nucleic acid fragment recovery system was employed to cut and recycle the large fragments. After purification, both ends of the DNA fragment were repaired and the addition A reaction was performed. Subsequently, the sequencing connector was connected, and finally Qubit was adopted to perform the precise detection of the constructed DNA library. The sequencing process was executed on R9.4 flow cells with GridION X5. For the SV phasing pipeline, reads were mapped to the human reference genome (GRCh37/hg19) with NGMLR, followed by sensitive SV predictions based on Sniffles. SV was annotated when the overlap degree between sequencing data and public database was greater than or equal to 50% (the distance between INS and INS of public database shall be less than or equal to 1000 bp). Moreover, such databases as 1000 genome phase3, DGV gold standard CNV, dbVar nstd37 and Decipher were utilized during the analysis. According to the phenotype of this disease, OMIM, HPO, Clinvar and other databases were searched with a view to identifying disease-related genes.

### Confirmatory Breakpoint PCR Primer Design and Sanger Sequencing Validation

The 600 kb sequences flanking the assumed breakpoints were searched from the UCSC genome website (GRCh37/hg19). Primer3 software and PrimerBank website were adopted to design primers for reference sequences and balanced reciprocal translocation breakpoint junction sequences. PCR was conducted according to the recommended protocols of manufacturers (RR001A; Takara). PCR products of breakpoint junction sequences were visualized on a 1.5% agarose gel, which was used to analyze the bands of normal and derivative chromosomes. The accurate breakpoint positions were confirmed by mapping Sanger sequencing sequences to GRCh37/hg19 human reference genome by minimap2 (version 2.10) and BLAT. If breakpoints lead to gene disruption or fusion, these genes would be retrieved on the website Online Mendelian Inheritance in Man (OMIM).

### Blastocyst Biopsy and WGA

Standard procedures were conducted in the ART process. Briefly, pituitary desensitization was conducted with controlled ovarian hyper-stimulation (COH) based on individual situations. In general, metaphase II (MII) oocytes were generated by intracytoplasmic sperm injection (ICSI), and subsequently were cultured for 5–6 days into the blastocyst stage. In this study, the criteria for grading blastocysts was in line with the standards recommended by Schoolcraft *et al* (Schoolcraft et al., 1999). Approximately 3–10 trophectoderm cells were biopsied and subsequently put into PCR tubes. The multiple displacement amplification method was utilized for WGA (QIAGEN, Hilden, Germany). CCS was performed on all the biopsied blastocysts with SNP-array.

### Junction-Spanning PCR and Haplotype Linkage Analysis

Junction-spanning PCR was performed according to the recommended protocols of manufacturers (RR001A; Takara). Junction-spanning PCR primers were the same as confirmatory breakpoint PCR primers in blood above, which included primers for forward reference sequence, reverse reference sequence, forward breakpoint junction sequence and reverse breakpoint junction sequence. As described previously, the SNP microarray could generate genome-wide SNP genotypes. The haplotype was created with informative SNPs, including the translocation breakpoint regions, the whole translocation chromosomes and the corresponding normal homologous chromosomes in the couple, reference and embryos. An unbalanced embryo or family member of carriers was used as reference to establish haplotypes. Informative SNPs were selected based on the criteria of homozygous in the spouse and heterozygous in the patient. Moreover, SNPs of the patient’s parents or other family members shall be homozygous if these SNPs are regarded as references to establish haplotypes. The haplotypes of both the translocated chromosome and the homologous chromosome could delineate the recombination of the breakpoint region.

### Embryo Transfer, Prenatal Diagnosis and Postnatal Follow-Up

Selected euploid blastocysts were transferred into the uterus of patients either 5 days after ovulation during a normal menstrual cycle or 5 days after ovulation initiated by progesterone treatment. The majority of two blastocysts were transferred, and the single blastocyst transfer was recommended for the patient with well-cryopreserved embryos. The clinical pregnancy was diagnosed if an intrauterine gestational sac with a heartbeat was detected by ultrasound imaging 30–40 days after embryo transfer. Amniocentesis was administered in the second trimester for pregnant patients. To validate the PGT results, amniocentesis fluid samples of fetuses were utilized for karyotyping analysis.

## Results

### *In Vitro* Fertilization (IVF) and CCS Results

In this study, both patients with chromosomal translocations underwent two IVF cycles. Both patients had one cycle (Supplementary Table S1). The information and COH results of these patients are listed in Supplementary Table S1. CCS with SNP-array was performed on all the 12 biopsied blastocysts, and six blastocysts were diagnosed as unbalanced abnormalities related to translocation. Among six blastocysts analyzed to be chromosomal balanced, three blastocysts were diagnosed as carriers and the other three were diagnosed to be normal. The specific results are listed in Table 1. The bioinformatics pipeline incorporating several analytical tools is presented in Figure 1.

**TABLE 1:** Detailed genetic testing results of the biopsied blastocysts.

Patient	Number of biopsied blastocysts	Grade of blastocysts	Molecular karyotype	Status of balanced translocation			Karyotype of fetus amniotic fluid
				Sanger method	PGH method		
Patient 1	Embryo-1	5BB	1q44*1; 2q32.1q37.3*3	Unbalanced		Unbalanced	
	Embryo-2	5BC	1q44*3; 2q32.1q37.3*1	Unbalanced		Unbalanced	
	Embryo-3	5BB	(1–22)*1,(XN)*1	Carrier		Carrier	Not transplanted
	Embryo-4	5BB	(1–22)*1,(XN)*1	Normal		Normal	Not transplanted
	Embryo-5	5BC	(1–22)*1,(XN)*1	Normal		Normal	46, XN
	Embryo-6	5BC	(1–22)*1,(XN)*1	Normal		Normal	Not transplanted
Patient 2	Embryo-1	5BC	12p13.33p13.31*3; 14q23.3q32.33*1	Unbalanced		Unbalanced	
	Embryo-2	5BB	(1–22)*1,(XN)*1	Carrier		Carrier	46,XN,t(12; 14)(p13.31; q23.3)
	Embryo-3	5BB	12p13.33p13.31*3; 14q23.3q32.33*1	Unbalanced		Unbalanced	
	Embryo-4	5CB	12p13.31q24.33*1; 14q11.1q23.3*3	Unbalanced		Unbalanced	
	Embryo-5	5BB	(1–22)*1,(XN)*1	Carrier		Carrier	Not transplanted
	Embryo-6	5BB	12p13.33p13.31*1; 14q23.3q32.33*3	Unbalanced		Unbalanced	

### High-Resolution Mapping of Translocation Breakpoints With ONT Platform

**FIGURE 2:**
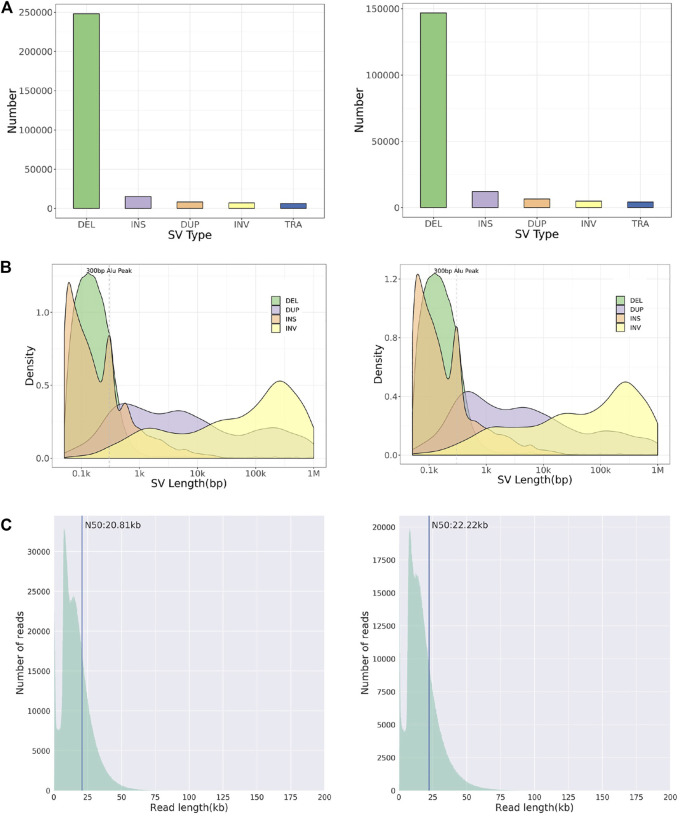
Chromosome structural aberration results of long-read Nanopore sequencing. Legend: The results of patient 1 (left) and 2 (right) are listed respectively. **(A)** Histogram of number of different types of SVs. **(B)** Length distribution of different types of SVs. **(C)** Diagram of reads length distribution. DEL, Deletion; INS, Insertion; DUP, Duplication; INV, Inversion; TRA, Translocation.

**FIGURE 3:**
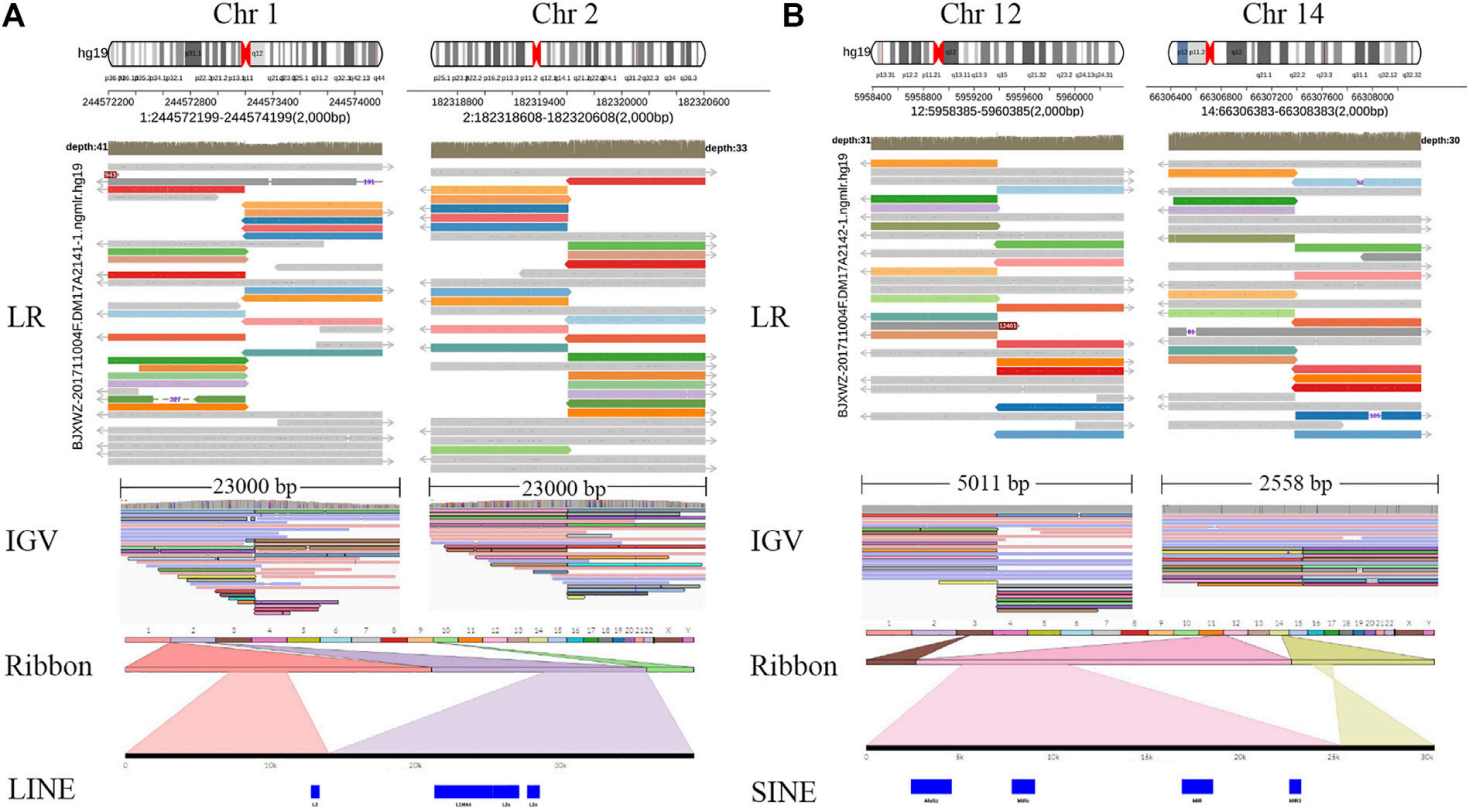
Chromosome rearrangement annotation and visualized analysis of Nanopore sequencing data. Legend: Translocation breakpoints detected by long-read Nanopore sequencing. Two cases, patients 1–2, were represented by **(A**,**B)** respectively. For each case, the top panel projected the chromosome pattern which translocation occurred, and LR alignments covering the breakpoint junctions. The panel in the bottom shows SR alignments (BWA-MEM; IGV-SR) and LR alignments (NGMLR; IGV-LR) across breakpoints using IGV. In Ribbon, chromosome 1 is shown in light orange and chromosome 2 in light purple, 22 recombined reads of chromosome 1 and 2 can be seen. Chromosome 12 is shown in pink, chromosome 14 is shown in yellow, 17 recombined reads of chromosome 12 and 14 can be seen. LR, long read; SR, short read; LINE, long interspersed nuclear elements; SINE, short interspersed nuclear elements; IGV, Integrative Genomics View; BWA-MEM, Burrows-Wheeler Aligner maximal exact matches.

Whole-genome and long-read sequencing analyses were performed on all subjects to obtain the accurate coordinates of breakpoints. Besides, low-coverage (∼20× coverage) Nanopore long-read sequencing was performed. In addition, SVs detected by Sniffles were screened out if the number of reads supporting SVs is greater than or equal to 2. The histogram number of SV statistics is presented in Figures 2A,B. The length distribution diagram of SVs between 30 bp and 1 Mbp is presented in Figure 2C. Further, 96.0 and 67.2 G bases were generated with the average length of 16,679 and 17,677 bp, respectively, for both patients (Table 2 and Supplementary Tables S2, S3). Mean mapping rates for both patients were larger than 96%. Additionally, chromosome translocations for both patients were found. In patient 1, a pair of translocation breakpoints were detected in 1q44 and 2q31. Translocation breakpoints spanning chr1:244,573,198-244,573,199 and chr2:182,319,607-182,319,608 were identified; twenty-two reads supported the translocation. The reliability was verified by Integrative Genomics View (IGV) and Ribbon (Figure 3A). While, in patient 2, a pair of translocation breakpoints were detected in 12p13 and 14q23. Translocation breakpoints spanning chr12:5,959,384-5,959,385 and chr14:66,307,382-66,307,383 were identified; 17 reads supported the translocation. The reliability was verified by IGV and Ribbon.

**TABLE 2:** Summary of breakpoint characterization results of the translocation by Nanopore and sanger sequencing.

Patient	Karyotype of peripheral blood cells	Depth (X)	Mapped sequencing reads, *n*	Mapped sequencing bases, *n*	Coverage rate (%)	Spanning break points reads, *n*	Break point position verified by sanger sequencing	SV segment length (%HLA)	Disrupted gene (break point)
Patient 1	46,XX,t(1; 2)(q44; q32.1), mat	32.01	5,570,349	93,191,761,872	96.83	22	244573198	4.68 Mb (1.88%)	ADSS (Intronic region)
						22	182319607–182319608	60.88 Mb (25.03%)	Intergenic region
Patient 2	46,XY,t(12; 14)(p13.31; q23.3), pat	22.39	3,661,947	64,963,815,620	96.39	17	5959384	5.96 Mb (4.45%)	ANO2 (Intronic region)
						17	66307374–66307382	41.04 Mb (38.23%)	Intergenic region

Reference sequence: GRCH37/hg19 reference genome.

By checking these breakpoints in the UCSC Genome Browser, two breakpoints were found inside introns of genes *ADSS* and *ANO2* in samples 1 and 2. Both breakpoints disrupted the normal gene structures, which resulted in the exchange of chromosomal segments, thereby potentially disturbing the gene function due to the movement of the gene fragment from one chromosome to another. In addition, among the four breakpoints, two breakpoints were identified inside the intergenic region. The breakpoint spanning chr2:182,319,607-182,319,608 (patient 1) was located between gene *LINC01934* and *ITGA4*. Besides, the breakpoint spanning chr14:66,307,382-66, 307, 383 (patient 2) was located between gene *FUT8* and *CCDC196*. Further, it could be found that no obvious deletion or duplication was caused by breakpoints. However, there was no obvious pathogenic phenotype of carriers from whom the above two samples were obtained, other than primary infertility. All these observations demonstrated the benefits of long reads for breakpoint characterization in genomic regions of low complexity.

### Validation of Breakpoints by Sanger Sequencing

**FIGURE 4:**
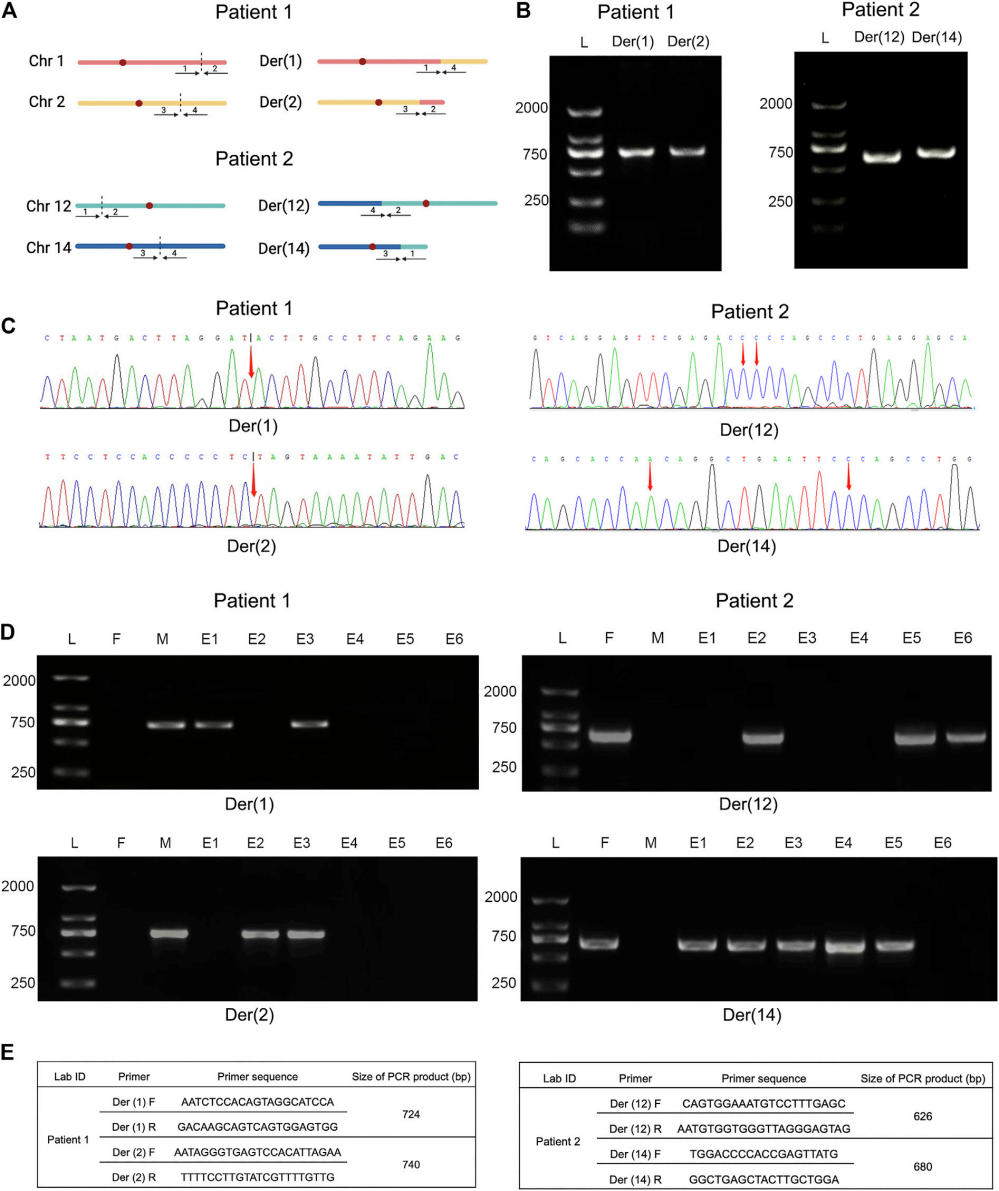
Comparative analysis of breakpoint PCR validation between Nanopore sequencing and embryo biopsies. Legend: **(A)** Ideogram of normal chromosomes and derivate chromosomes of patient 1 (left) and 2 (right). Dotted portion represents breakpoint identification. **(B)** PCR validation of translocation variants. PCR products of patient 1 (left) and 2 (right) were shown, respectively. **(C)** Schematic diagrams of precise breakpoints characteristics of patient 1 (left) and 2 (right) by Sanger sequencing. The coordinates of breakpoints were showed (red arrows). **(D)** Junction-spanning PCR and AGE validation of patient 1 (left) and 2 (right), along with biopsied embryos after PGT-SR cycles, respectively. For both individual, four fragments, two pairs of breakpoint junctions, i.e. der (1) and der (2), der (12) and der (14) were verified respectively. L, DNA ladder; M, mother; F, father; E, embryo; PCR, polymerase chain reaction; AGE, agarose gel electrophoresis. **(E)** Primers used for amplification of translocation breakpoints. F, forward primers; R, reverse primers; bp, base pairs.

To further verify translocation calls identified by Nanopore sequencing, PCR and Sanger sequencing were performed on the precise adjacent sequences of rearrangement breakpoints. The primer information is presented in Figure 4E. Primers for forward reference sequence, reverse reference sequence, forward breakpoint junction sequence and reverse breakpoint junction sequence were designed for each translocation breakpoint. An ideogram of normal chromosomes and derivate chromosomes of patients 1 and 2 was established. For patients 1 and 2, both PCR products and breakpoint junction sequences were observed, which validated the two translocation variations. In addition, the target PCR bands reflecting translocated chromosomes could be found in patients 1 and 2. The length of PCR products was in line with the expected product size. The precise breakpoint positions detected by Sanger sequencing for all rearranged fragments are shown in Table 2 and Figure 4C. The results of sequencing in these breakpoint regions illuminated the complexity of human genome shuffling.

### Embryo Biopsies, Junction-Spanning PCR and Haplotype Linkage Analysis

To evaluate the feasibility of Nanopore sequencing in PGT-SR, junction-spanning PCR analysis was performed on 12 WGA products of 12 euploid embryos, in an attempt to identify the noncarrier embryos and carrier embryos based on translocation breakpoints. For trophectoderm biopsies, it was found that there was 100% concordance (12/12) of breakpoint PCR for patients 1 and 2. The results of junction-spanning breakpoint PCR are presented in Table 3 and Figure 4D. These findings indicated that these three blastocysts were reciprocal translocation carriers, the other three were normal noncarriers, and the remaining six were unbalanced embryos. The designed primers’ information on the reference sequences and balanced reciprocal translocation breakpoint junction sequences is presented in Figure 4E.

**TABLE 3:** Detailed junction-spanning PCR results of the biopsied blastocysts.

Patient	Number of biopsied blastocysts	Junction-spanning PCR results		Comprehensive result
		Der (1)	Der (2)	
Patient 1	Embryo-1	Positive	Negative	Unbalanced
	Embryo-2	Negative	Positive	Unbalanced
	Embryo-3	Positive	Positive	Translocation carrier
	Embryo-4	Negative	Negative	Normal
	Embryo-5	Negative	Negative	Normal
	Embryo-6	Negative	Negative	Normal
		Der (12)	Der (14)	
Patient 2	Embryo-1	Negative	Positive	Unbalanced
	Embryo-2	Positive	Positive	Translocation carrier
	Embryo-3	Negative	Positive	Unbalanced
	Embryo-4	Negative	Positive	Unbalanced
	Embryo-5	Positive	Positive	Translocation carrier
	Embryo-6	Positive	Negative	Unbalanced

Meanwhile, the haplotype analysis approach of breakpoints was adopted. The detailed haplotype phasing results of the biopsied blastocysts are listed in Table 4. Informative SNPs were successfully generated adjacent to the breakpoint regions, which enabled the detection of the rearranged chromosomes and the corresponding normal homologous chromosomes in samples 1 and 2 (Table 5). Subsequently, haplotypes were employed to discriminate between embryos with balanced translocation chromosomes and those with structurally normal chromosomes through linkage analysis. A total of 12 embryos were detected. Among them, three embryos were detected with normal karyotypes and three were with balanced translocation karyotypes, the results of haplotype analysis were in line with PCR analysis as expected.

**TABLE 4:** Detailed haplotype phasing results of the biopsied blastocysts.

Patient	Number of biopsied blastocysts	Reference of haplotype phasing	Haplotype results of breakpoint regions (2 Mb)		Comprehensive result
Patient 1		Grandmother	1q44	2q32.1	
	Embryo-1		Derivative	Normal	Unbalanced
	Embryo-2		Normal	Derivative	Unbalanced
	Embryo-3		Derivative	Derivative	Translocation carrier
	Embryo-4		Normal	Normal	Normal
	Embryo-5		Normal	Normal	Normal
	Embryo-6		Normal	Normal	Normal
Patient 2		Grandfather	12p13.31	14q23.3	
	Embryo-1		Normal	Derivative	Unbalanced
	Embryo-2		Derivative	Derivative	Translocation carrier
	Embryo-3		Normal	Derivative	Unbalanced
	Embryo-4		Normal	Derivative	Unbalanced
	Embryo-5		Derivative	Derivative	Translocation carrier
	Embryo-6		Derivative	Negative	Unbalanced

**TABLE 5:** Summary of informative SNPs used to establish the haplotypes of the diploid blastocysts.

Patient	Embryo number	Breakpoint position	The total number of informative SNPs in breakpoint (2 Mb)	The total number of informative SNPs in chromosome	The number of recombination informative SNPs	The location of recombination in chromosome	Whether recombination happens in the breakpoint regions?
Patient-1	Embryo-3	Chr1:244573198	18	1694	127	1:1-12186274 (p36.33p36.22)	No
					423	1:48083274-107951986 (p33p13.3)	
		Chr2:182319607	4	1770	341	2:27807350-65522654 (p23.3p14)	No
					328	2:193324405-234037544 (q32.3q37.1)	
	Embryo-4	Chr1:244573198	18	1681	828	1:34419776-163565845 (p35.1q23.3)	No
					226	1: 205474838-231902291 (q32.1q42.2)	
					44	1:245135196-249250621 (q44)	
		Chr2:182319607	3	1722	196	2:102068311-131602187 (q11.2q21.1)	No
					335	2:193324405-243199373 (q32.3q37.3)	
	Embryo-5	Chr1:244573198	18	1666	503	1:38670335-82095444 (p34.3p31.1)	No
					264	1:156883028-201751539 (q23.1q32.1)	
		Chr2:182319607	4	1750	648	2:1-66217787 (p25.3p14)	No
					24	2:240229300-243199373 (q37.3)	
	Embryo-6	Chr1:244573198	15	1625	649	1:1-65798457 (p36.33p31.3)	No
		Chr2:182319607	4	1662	366	2:23021659-64943105 (p24.1p14)	No
					171	2:222699721-243199373 (q36.1q37.3)	
Patient-2	Embryo-2	Chr12:5959384	22	890	14	12:1-2019430 (p13.33)	No
					812	12:25776583-133851895 (p12.1q24.33)	
		Chr14:66307374	20	671	94	14:1-29117385 (p13q11.2)	No
	Embryo-5	Chr12:5959384	25	997	30	12:1-3538270 (p13.33p13.32)	No
					679	12:43737510-133851895 (q12q24.33)	
		Chr14:66307374	20	722	87	14:96027947-107349540 (q32.13q32.33)	No

### Clinical Outcomes and Validation of Efficacy

Two euploid embryos were selected and transferred into the uterus in both patients. Subsequently, both patients were pregnant, amniocentesis was performed and two healthy babies were delivered successfully. It could be verified that there was 100% consistency between the bioinformatics analysis results of PGT and the cytogenetic results. Newborns born in PGT-SR cycles were all healthy. Besides, the existence of the rearranged chromosomes was also detected through linkage analysis. Therefore, the predicting accuracy of the proposed method was further demonstrated by the above-mentioned results.

## Discussion

Despite the fact that many carriers with balanced translocation can achieve successful live births through PGT treatment, these carriers may transmit the parental translocation aberrations to their offspring. In this situation, their children may also suffer from infertility in the future. Moreover, the genetic risk induced by *de novo* chromosomal translocation usually cannot be assessed in phenotypically normal balanced translocation carriers. There are still challenges for reliable molecular breakpoint detection of carrier embryos from those with normal karyotype due to short read lengths and application limitations.

Numerous techniques are being developed to facilitate breakpoint deciphering during PGT-SR cycles. The resolution ratio of the FISH technique is approximately 100 kilobase to 1 mega base in size (Cui et al., 2016). However, due to the fact that FISH is restricted by the requirements of specific fluorescent probes, complex procedures, and ambiguous fluorescence signals, it is necessary to develop more novel methods to accomplish a precise translocation breakpoint analysis (Wilton et al., 2009). Recently, Treff *et al.* reported that SNP genotype could be employed to distinguish two normal human embryos from 46 embryos (Treff et al., 2011b). Besides, the qPCR-based comprehensive chromosome analysis can be conducted to detect chromosomal aberrations in PGT cycles (Cimadomo et al., 2018b). With the significant advancement of sequencing technologies, NGS is becoming a potentially applicable approach for breakpoint detection and chromosomal rearrangement identification in clinical settings (Tan et al., 2014; Trautmann et al., 2019). In fact, the transition from multiplex PCR to universal processes with WGA, followed by SNP array or NGS marks a significant advancement over the last decade. The waiting period of subjects has been substantially reduced as a result of reduced lab workload and omission of customized preclinical inspections (De Rycke and Berckmoes 2020). Genotype-based NGS has been regarded as a promising platform for future PGT since NGS allows an all-in-one solution for chromosome aberration assessment (Popovic et al., 2020). However, short-read sequencing approaches are still restricted by their unfavorable sensitivity (only 10–70% of chromosome rearrangements can be detected) (Huddleston et al., 2017) and very large false positive rates (Teo et al., 2012; Sudmant et al., 2015). Moreover, on the ground that rearranged breakpoints typically occurred in complex regions, such as GC-bias regions, AT-rich short tandem-repeat regions and long low-copy repeat (LCR) regions, complex segments may be prone to be misinterpreted and not all translocation breakpoints can be mapped due to the short-read length of NGS (Wang et al., 2017).

Currently, new PGT-SR methods, e.g. PGH, are being developed, and chromosomal haplotyping has been effectively utilized to distinguish normal human embryos from translocation carriers (Xu et al., 2017; Zhang et al., 2017). The SNP haplotyping approach depends on the analysis of informative SNP markers to flank breakpoints. As for SNP-array techniques, however, it is necessary to take aneuploid embryos with the translocated chromosome or samples from the carriers’ parents as references. Nowadays, Nanopore long-read sequencing is becoming a technically powerful and a clinically practical approach. Long reads-based sequencing could significantly promote the development of the phasing of chromosome aberrations at an unprecedented scale. LRS outperforms SRS in the determination of complex rearranged regions (Lu, Giordano, and Ning 2016). It has been indicated that a portable Nanopore-based sequencer can be employed to perform fast preimplantation genetic diagnosis onsite on five samples within 2 h (Wei et al., 2018). 10–30× coverage of long reads was reported to cover ∼80% of full types of SV calls with ∼80% precision or even higher (Kato et al., 2012). In this study, a reliable strategy was developed to distinguish noncarrier from carrier embryos in PGT-SR cycles for balanced translocation. Based on the breakpoint analysis of the blood from translocation carriers through Nanopore sequencing and Sanger sequencing, junction-spanning PCR and haplotype linkage analysis were then performed in embryos, the results of which demonstrates that this present method can be employed to distinguish translocation-free euploid embryos in PGT-SR cycles.

To the best of our knowledge, this is one of the first studies exploring the detection of normal karyotype in embryos. Besides, single-molecule long-reading sequencing is creatively introduced into PGT-SR cycles for clinical application. Compared with existing methods, there are several distinct advantages in this approach. First, in comparison to existing technologies, Nanopore long-read sequencing could be employed to decipher high-resolution breakpoint mapping directly and localize the disrupted genes precisely. Second, Nanopore long-read sequencing provides an average length of 10 kb, which significantly increases opportunities for mapping the overlapped breakpoints of chimeric reads. Third, it takes only 1.5 h for Nanopore sequencing library preparation, instead of costing days setting up the NGS library. Fourth, the cost of detecting breakpoint with Nanopore sequencing in blood samples is close to that with NGS. It is reported that 10× coverage of the whole genome can be detected with 10–15 MinION flowcells at a cost of $1000–$2000 in 7 days of sequencing duration (Middelkamp et al., 2017). Nonetheless, there are still some drawbacks in this approach. Firstly, the method is not feasible for Robertsonian translocation carriers, due to the fact that their breakpoints are in the highly repetitive centromeric regions of subtelocentric chromosomes. Secondly, the results of breakpoint PCR may be affected by the location of breakpoints.

Carriers with structural rearrangements suffer a high risk of producing unbalanced gametes due to aberrant separation during meiosis. A 1:1 rate of noncarrier to carrier embryos can be determined among all the biopsied embryos in this study. This result is similar to a previous study, in which 49% of 126 human balanced translocation embryos have been diagnosed as noncarrier embryos and the other 51% have been diagnosed as carrier embryos (Treff et al., 2016). The frequency of unbalanced chromosome recombinants seems to be affected by multiple factors, such as the coordinates of breakpoints, the size of the centric/translocated fragment, the region involved, and the chromosome affected as reported (Zhang et al., 2018; Mateu-Brull et al., 2019). Although there are different segregation patterns, the overwhelming meiotic segregants are 2:2 adjacent-1, followed by 2:2 alternate. Among all possible gametes, only two from alternate separation patterns have normal or balanced chromosomes, and the remaining gametes are genetically unbalanced. According to the theoretical basis of segregation theory, the incidence of normal or balanced gametes is relatively low (Scriven et al., 1998). It has been found that chromosome rearrangement could interfere with the correct separation of other chromosomes by disrupting the arrangement of chromosomes during meiosis I. Additionally, it has been reported that there is a trend of a higher rate of abnormal segregation patterns with a smaller size of translocated segments as well as smaller chromosomes has been reported (Jalbert et al., 1980). Besides, quadrivalents with acrocentric chromosomes are related to a higher percentage of unbalanced adjacent-1 segregants, and asymmetric quadrivalents are related to a higher incidence of adjacent-2 segregants (Zhang et al., 2018; Mateu-Brull et al., 2019).

The detected breakpoints in chromosomes can lead to gene interruption, and gene fusion, and exert position effect at the breakpoint junctions. Although the transcriptional outcomes of most chromosome structural aberrations have not been investigated, there is a risk to break genetic rules for rearrangement-mediated breakpoints that fuse or disrupt relevant genes (Canela et al., 2017). Due to the fact that the included patients have normal phenotypes, the interruption may not produce a pathogenic phenotype or may cause an undetectable phenotype. In this study, insertion sequences of less than 40 bases, which are induced by fallible non-homologous end-joining (NHEJ) or microhomology-mediated break-induced replication (MMBIR) after DNA double strand breaks down at the conjunction with translocation (Smith et al., 2005), are detected adjacent to the breakpoint junctions in all four derivative chromosomes. The majority of the constitutional translocations are not recurrent, and microhomology is a typical characteristic at breakpoint junctions. Moreover, although the sequence at breakpoints can be modified by resection, insertion, and translocation, it has been suggested in recent studies that mutation may also occur in sequences from breakpoint junctions (Weckselblatt and Rudd 2015). Chromosome structural rearrangements may also impose position effects at the breakpoint junctions, which could diversify the expression of related intact genes. It has been reported that in the translocation between chromosomes 11 and 22, the aberrant nuclear localization of translocated segments contributes to the altered expression of several genes on chromosomes (Harewood et al., 2010). Further, it is necessary to investigate the phenotypes of cis and trans position effects of structural aberrations when WGS breakpoint mapping fails to pinpoint genes associated with diseases (Gilissen et al., 2014).

In summary, techniques for breakpoint analysis based on long-read Nanopore sequencing are currently being investigated. Based on the clinical validation, this method has been demonstrated to have favorable feasibility. The strategy proposed in this study may provide a way of selecting embryos with normal karyotypes during PGT-SR treatment cycles. Junction-spanning PCR in embryos proves to be alternative, especially for those without a reference. The sensitivity and specificity of this strategy require to be further verified due to a relatively small sample size.

## Data Availability

The original contributions presented in the study are included in the article/Supplementary Material, further inquiries can be directed to the corresponding authors.
