# Supplementary material for: Identifying Balanced Chromosomal Translocations in Human Embryos by Oxford Nanopore Sequencing and Breakpoints Region Analysis
Source: Front Genet. 2022 Jan 18;12:810900. doi: 10.3389/fgene.2021.810900 (PMC8804325; doi:10.3389/fgene.2021.810900)
Supplement: Supplementary file 1 [file Table3.DOC]

| **Supplement Table S3. The quality of ONT sequencing** | | | | | | | |
| --- | --- | --- | --- | --- | --- | --- | --- |
| Patient | Total bases | Reads num | Mapping rate | Mean length | Max length | N50 | Depth |
| Patient 1 | 96,018,388,215 | 5,756,740 | 96.83% | 16,679 | 177,022 | 20,808 | 32.01 |
| Patient 2 | 67,160,078,051 | 3,799,096 | 96.39% | 17,677 | 182,031 | 22,216 | 22.39 |
