# Supplementary material for: Identifying Balanced Chromosomal Translocations in Human Embryos by Oxford Nanopore Sequencing and Breakpoints Region Analysis
Source: Front Genet. 2022 Jan 18;12:810900. doi: 10.3389/fgene.2021.810900 (PMC8804325; doi:10.3389/fgene.2021.810900)
Supplement: Supplementary file 2 [file Table1.DOC]

| **Table S1. The characteristics of the patients in this study** | | | | | | | | | |  |
| --- | --- | --- | --- | --- | --- | --- | --- | --- | --- | --- |
| Patient ID | Maternal age | Karyotype ^a^ | Reason for karyotyping | The number of | | The number of | The number of | The number of | The number of | |
|  | /Paternal age |  |  | oocytes retrieved | | mature oocytes (MII) | fertilized oocytes | D3 oocytes | biopsied blastocysts | |
| Patient-1 | 25/26 | 46,XX,t(1;2)(q44;q32.1), mat | Repeated miscarriage | 20 | 19 | | 17 | 14 | 6 | |
| Patient-2 | 25/25 | 46,XY,t(12;14)(p13.31;q23.3), pat | Repeated miscarriage | 13 | 12 | | 8 | 8 | 6 | |
| ^a^ The karyotypes were identified by peripheral blood cells. | | | | | | | | | | |
