# Supplementary material for: Identifying Balanced Chromosomal Translocations in Human Embryos by Oxford Nanopore Sequencing and Breakpoints Region Analysis
Source: Front Genet. 2022 Jan 18;12:810900. doi: 10.3389/fgene.2021.810900 (PMC8804325; doi:10.3389/fgene.2021.810900)
Supplement: Supplementary file 3 [file Table2.DOC]

| **Supplement Table S2. SV detection and statistics** | | | | | | |
| --- | --- | --- | --- | --- | --- | --- |
| Patient | DEL | DUP | INS | INV | TRA | Total |
| Patient 1 | 248,035 | 8,264 | 15,094 | 7,243 | 6,291 | 284,927 |
| Patient 2 | 146,784 | 6,550 | 12,115 | 4,906 | 4,348 | 174,703 |

DEL: Deletion; INS: Insertion; DUP: Duplication; INV: Inversion; TRA: Translocation
